# Representing Dental Caries and Dysbiosis within the Oral Microbiome in the Oral Health and Disease Ontology

**DOI:** 10.21203/rs.3.rs-7265626/v1

**Published:** 2025-09-19

**Authors:** William D. Duncan, Amarpreet Sabharwal, Alexander D. Diehl, Nivedita Dutta, Matthew Diller, Marcin P. Joachimiak, Gopikrishnan M. Chandrasekharan

**Affiliations:** University of Florida College of Dentistry; Western University; University at Buffalo; Self-Employed Clinician and Clinical Researcher; National Library of Medicine, National Institutes of Health; Lawrence Berkeley National Laboratory; Indiana University Indianapolis

**Keywords:** oral microbiome, microbial dysbiosis, dental caries, Oral Health and Disease Ontology

## Abstract

**Background.:**

Dental caries is an oral health condition in which cariogenic bacteria demineralize and decay teeth. It arises due to interaction between the host, environment, and oral microbiome. Current terminologies and ontologies, however, do not accurately represent the important role that the microbiome has in the formation of carious lesions. Rather, they focus on the anatomical features of carious lesions and often obfuscate the distinctions between dental caries as a disease affecting a tooth, as lesions that are produced because of the disease, and as lesions produced as a result of dysbiosis in the oral microbiome. To capture the current state of evidence and provide flexibility for evolving literature on host-environment-microbiome interactions, there is a need to revise and expand the ontological framework for dental caries.

**Results.:**

Several established terminologies and ontologies were reviewed for terms used to represent dental caries and the oral microbiome. We found that they either did not represent or misrepresented the current scientific understanding of caries and its relation to the microbial dysbiosis. As a result of these deficiencies, we added terms and relations to the Oral Health and Disease Ontology (OHD) that more accurately represent how oral microbial dysbiosis influences the development of dental caries.

**Conclusions.:**

The Oral Health and Disease Ontology is an advance over existing ontologies for representing the impact of oral microbial dysbiosis on dental caries. It provides a semantic framework that better serves the needs of cariology researchers and can more easily incorporate new oral microbiome findings.

## 1. Background

Dental caries is a common dental condition [1] that arises due to interaction between the host, environment, and oral microbiome [2]. A considerable proportion of the oral microbiome is found attached to oral hard and soft tissues in a complex but reproducible arrangement, and embedded in an extracellular matrix, called oral biofilm [3]. Dental caries lesions (cavities) result from a complex interplay between the presence of cariogenic bacteria such as *Streptococcus mutans* and *Streptococcus sorbinus* in oral biofilm and the availability of a carbohydrate-rich diet. The metabolic end products of this interplay between specific microbiota and the dietary source are acids capable of demineralizing dental hard tissues [3, 4]. Balance between risk factors (such as reduced salivary flow, frequency and composition of carbohydrate rich foods) and protective factors (such as good oral hygiene and regular dental care visits) determines the extent and severity of dental caries [4].

Several hypotheses attempt to explain the microbial etiopathogenesis of dental caries. The non-specific plaque (biofilm) hypothesis and the specific plaque (biofilm) hypothesis present contrarian views on the role of cariogenic microbiota in oral biofilm. The non-specific plaque hypothesis posits that there is a linear relationship between the quantity of dental biofilm and the resulting dental caries with the inherent assumption that all microbes are participants in disease causation [4, 5]. As more data on the functional heterogeneity of microbes in the dental biofilm have become available, this viewpoint has been refined. The specific plaque hypothesis posits that certain microbiota are crucial in the causation of dental caries (cariogenic bacteria) [4, 6]. An additional hypothesis, the ecological plaque hypothesis, emphasizes the role of environmental factors and considers them to be driving factors for the selection of bacteria in a biofilm population [7, 8]. Conversely, there will be bacteria that do not thrive in particular environments and as such the environment controls the selection of a disease-specific biofilm. Similarly, in the extended ecological plaque hypothesis, it is proposed that pathogenic microbiota, selected by the environment, can further shape the disease-specific biofilm and therefore, the environment and microbiota act synergistically to shape the disease process [9]. This evolution of theories to explain the etiopathogenesis of dental caries emphasizes the role of three elements: host, environment, and microbes.

Dental caries management in routine clinical practice includes control of active carious lesions by removal of infected and affected tooth structures, restoration of carious defects, and prevention regimens to eliminate or reduce the development of new carious lesions [10]. Current terminologies and ontologies, however, do not accurately represent the important role that the microbiome has in the formation of carious lesions. Rather, they focus on the anatomical features of carious lesions, such as being demineralized or cavitated, and often obfuscate the distinctions between dental caries as a disease affecting a tooth, as lesions that are produced as a result of the disease, and as lesions produced as a result of dysbiosis in the oral microbiome. As our understanding of caries associated with the dysbiotic microbiome develops, precision approaches to modulate host immunity and microbiome will certainly follow [11]. In this regard, there has been considerable progress in *in vitro* systems and *in vivo* animal models. Directed approaches to modulate the dysbiotic microbiome may include vaccines, probiotics, and small molecules (synthetic or natural) [12-15]. To capture the current state of evidence and provide flexibility for evolving literature on host-environment-microbiome interactions, there is a need to revise and expand the ontological framework for dental caries. In this manuscript, we review how dental caries is classified in several prominent terminologies and ontologies, and based on our review, we discuss the modifications made to the Oral Health and Disease Ontology (OHD) [16] to more accurately represent the scientific understanding of the role the oral microbiome has in dental caries.

## 2. Materials and Methods

Our research team, which included two experienced ontologists, a microbiome researcher, and three dentists, analyzed several prominent biomedical terminologies and ontologies. To find these ontologies, we searched Google Scholar and PubMed for relevant articles, and we searched the ontology browsing services Ontology Lookup Service [17], Bioportal [18], Ontobee [19], and Aber-OWL [20] for relevant ontologies.^[1]^

Our searches in Google Scholar and PubMed did not find any ontologies for relating dental caries to oral microbial dysbiosis. On Google Scholar, we used the query ((ontology and caries and microbiome) -"gene ontology" -"Antibiotic Resistance Ontology"). In order to exclude a large number of false positives, we had to exclude the terms “gene ontology” and “Antibiotic Resistance Ontology”. The query returned 259 results, but these articles mentioned ontologies in context of data analysis and were not about developing an ontology for representing how dental caries is related to imbalances within the oral microbiome. On PubMed, we used the query (ontology and caries and microbiome). This returned five results in the last ten years. Again, these articles were not about developing an ontology for relating dental caries and oral microbial dysbiosis.

On the ontology services we searched for the terms ‘caries’ or ‘dental caries’. This returned 25 ontologies on the Ontology Lookup Service, 31 ontologies on Bioportal, 12 ontologies on Ontobee, and 23 ontologies on Aber-OWL. After reviewing the results, we focused on analyzing the Systematized Nomenclature of Medicine - Clinical Terms (SNOMED CT) [21], The International Classification of Diseases, 11th ed. (ICD-11) [22], Medical Subject Headings (MeSH) [23], NCI Thesaurus (NCIt) [24], Human Disease Ontology (DO) [25], Monarch Disease Ontology (MONDO) [26], and the Human Phenotype Ontology (HPO) [27]. Several ontologies were excluded from our analysis because they were very similar, and some cases had identical branches, to the other select ontologies. ICD-11 was selected because of the substantial role it has in providing medical diagnosis codes, and MeSH was chosen because of its importance for searching PubMed. After identifying the deficiencies in the selected terminologies and ontologies, we added terms and relations to the Oral Health and Disease Ontology (OHD) that more accurately represent how oral microbial dysbiosis influences the development of dental caries.

## 3. Results

Our analysis of the selected ontologies (below) determined that they did not accurately represent the complex multi-factor etiology of dental caries: interactions of the host, environment and microbiome. Based on these deficiencies, in this iteration, we expanded the OHD to provide a more comprehensive representation of the role of the microbiome in dental caries. The emphasis on microbiome is considered first due to the rapid development of knowledge in this field. Note, to improve readability, **bold** text is used for classes and *italics* text is used for relations.

### 3.1 Systematized Nomenclature of Medicine - Clinical Terms (SNOMED CT)^[2]^

SNOMED CT is a comprehensive terminology and covers a wide range of clinical concepts, such as diseases, symptoms of disease, medical procedures, and clinical findings. Its structure includes hierarchical relationships (parent-child/subtypes), associative relationships, and qualifiers that provide additional context. Relationships between a concept and its parent (or more general) concept are denoted using an *Is-a*^[3]^ property. For example, the axiom **Disease**^[4]^ *Is-a* **Clinical finding**^[5]^ defines the concept **Disease** as a type of (i.e., a child concept) of **Clinical finding**.

**Figure 1:**
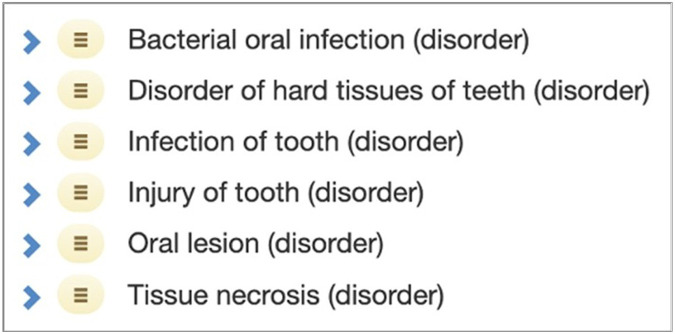
The parent concepts of **Dental caries** in SNOMED CT.

In SNOMED CT, the **Dental caries**^[6]^ concept has multiple parents: **Bacterial oral infection**^[7]^, **Disorder of hard tissues of teeth**^[8]^, **Infection of tooth**^[9]^, **Injury of tooth**^[10]^, **Oral lesion**^[11]^,and **Tissue necrosis**^[12]^ (see Figure 1).

There are a few important issues to discuss with this classification. The first is that the logical structure of SNOMED CT defines **Dental caries** as the conjunction (not disjunction) of all its parents. This polyhierarchy leads to false assertions. For instance, it entails that the concept **Enamel caries** (a child concept of **Dental caries**), is defined as both a type of **Oral lesion** and a type of **Infection of tooth**. While the classification of**Enamel caries** as a type of **Oral lesion** may be reasonable, it cannot be an **Infection of tooth**. Enamel does not consist of living cells, and thus cannot be infected. Moreover, a similar mistake holds in regards to **Dental caries** as a type of **Tissue necrosis**. Since enamel is inorganic, it does not undergo necrosis.

Second, SNOMED CT conflates the pathological processes that produce lesions produced with the lesions themselves. The processes that produce lesions begin before visible and tactile lesions are present on the tooth surface following bacterial demineralization. Restoring the tooth surface with a dental restorative material after removing the caries lesion may restore the function of the tooth. However, we cannot conclude that the processes that caused the lesions have been abated. The microbial and environmental factors that caused the carious lesion may still be present.

Finally, SNOMED CT does not elaborate on how dysbiosis of the dental biofilm microbiome results in bacterial demineralization. It does contain the concept **Demineralization of tooth**^[13]^ as a separate disorder, but the concept is not related to the oral microbiome. At present, the only concept concerning dysbiosis is **Intestinal dysbiosis**^[14]^.

### 3.2 The International Classification of Diseases, 11th ed. (ICD-11)

ICD-11 is a hierarchical classification maintained by the World Health Organization (WHO) for coding diseases, conditions, and other health-related issues for statistical and billing purposes. ICD-11 uses an alphanumeric coding system to represent diseases and health conditions which are further divided into blocks, categories and subcategories to provide an increasing level of specificity.

Similar to SNOMED CT, ICD-11 classifies **Dental caries**^[15]^ under **Diseases of hard tissues of teeth**^[16]^. However, neither ICD-11 nor its previous iterations classifies dental caries as a disease in which dysbiosis plays a role. Terms like ‘microbiome’ and ‘dysbiosis’ are missing from ICD-11. Given the origins of ICD-11 and its inability to relate terms in different branches in its hierarchy, this is not particularly surprising. However, it does demonstrate that ICD-11 is not currently structured for use in analysis on oral microbiome associated diseases, and this is an important oversight.

### 3.3 Medical Subject Headings (MeSH)^[17]^

**Figure 2:**
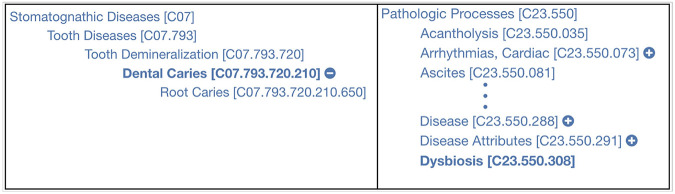
The parent concepts of **Dental caries** and **Dysbiosis** in MeSH.

MeSH is a terminology and hierarchical classification system developed by the National Library of Medicine (NLM) to function as a thesaurus and provide context to knowledge in resources such as PubMed. Similar to ICD-11, MeSH uses a hierarchical structure in which **Dental caries**^[18]^ is classified as a type of **Tooth Demineralization**^[19]^, and **Dysbiosis**^[20]^ is a subtype of **Pathologic Processes**^[21]^ (see Figure 2).

We find MeSH’s classification of **Dental Caries** and **Dysbiosis** problematic in the following ways. First, it is not clear if **Dental Caries** is a process or physical entity. The MeSH hierarchy classifies **Dental Caries** as the process of damaging a tooth. **Dental Caries** is a child term of **Stomatognathic Diseases**^[22]^, and as seen in the **Dysbiosis** hierarchy, a **Disease** is a child of **Pathological Process**. However, the scope note for **Dental Caries** suggests that **Dental Caries** is a cavity in the tooth^[23]^:

Localized destruction of the tooth surface initiated by decalcification of the enamel followed by enzymatic lysis of organic structures and leading to cavity formation. If left unchecked, the cavity may penetrate the enamel and dentin and reach the pulp.

Moreover, **Caries Lesions, Dental Cavity**, and **Dental White Spots** are included as entry terms (i.e, synonyms, near-synonyms, alternate forms, and other closely related terms)^[24]^ for **Dental Caries**. We recognize that in natural language, a clinician may, depending on context, use the expression ‘dental caries’ to refer to the process of a tooth decaying or a cavity resulting from such decay. The ambiguity, however, is confusing for purposes of defining dental caries in an ontology.

Second, we find similar ambiguities in MeSH’s classification of **Dysbiosis**. As previously mentioned, **Dysbiosis** is a child term of **Pathological Process**, but the scope note suggests that it is a physical state of the microbiome^[25]^:

Changes in quantitative and qualitative composition of microbiota. The changes may lead to altered host microbial interaction or homeostatic imbalance that can contribute to a disease state often with inflammation.

Again, this ambiguity is not constructive for developing an ontology.

Finally, even if we accept the ambiguities of MeSH’s **Dental Caries** and **Dysbiosis** terms, there is not a clear way to relate these terms in the MeSH’s classification system. At best, MeSH’s annotations provide some information about how terms are related. For instance, **Dental Caries** includes a ‘See also’ annotation to **Cariogenic Agents**, but neither **Cariogenic Agents** or other annotations connect **Dental Caries** to **Dysbiosis**.

### 3.4 NCI Thesaurus (NCIt)^[26]^

The NCIt is an ontology and controlled vocabulary developed by the National Cancer Institute for the primary purpose of providing concepts for describing cancer-related research and related domains. In NCIt, **Caries**^[27]^ is a child concept of **Oral Cavity Finding**^[28]^. We find NCIt’s representation of **Caries** deficient in two ways. First, NCIt’s definition of **Caries** has semantic issues when both it and **Caries’** child concepts are considered together. **Caries** is defined as:

The decay of a tooth, in which it becomes softened, discolored, and/or porous.

This definition connotes **Caries** as a process during which the tooth is decayed, but the child concept **Secondary Caries**^[29]^ is defined as kind of carious lesion (i.e., a physical structure) :

A carious lesion adjacent to an existing restoration.

This incongruence between parent and child concepts has the potential to cause semantic interoperability issues. Moreover, **Cavities** is given as a synonym for **Caries**, a cavity is not a process. Second, although the NCIt has an **Oral Microbiome**^[30]^concept, it does not relate it to **Caries**.

### 3.5 Open Biological and Biomedical Ontologies (OBO) Foundry and Dental Caries

The Open Biological and Biomedical Ontologies (OBO) Foundry [28] is a collaborative effort to create and maintain well-structured and logically consistent ontologies using defined rules and principles such that these ontologies are modular and interoperable to facilitate data exchange among various biomedical and biological research communities. In the OBO Foundry, each ontology focuses on particular domains, such as disease or anatomy, with the aim of having other OBO Foundry ontologies reuse concepts from other member ontologies. The main OBO Foundry ontologies representing dental caries are the Human Disease Ontology (DO), Monarch Disease Ontology (MONDO), Human Phenotype Ontology, and Oral Health and Disease Ontology (OHD). Among these ontologies, the OHD is the only ontology primarily focused on the oral health domain, whereas DO, MONDO, and HPO represent diseases and disease-related entities in multiple domains. In our analysis we found the representation of dental caries deficient in DO, MONDO, and HPO. Based on these deficiencies, we modified the OHD to address these shortcomings.

#### 3.5.1 Ontology for General Medical Science (OGMS)^[31]^

**Figure 3:**
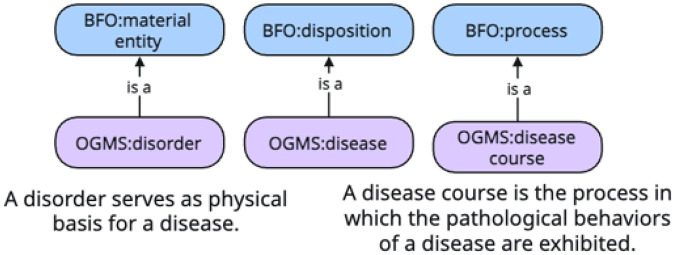
The OGMS **disorder, disease**, and **discourse course** terms.

The Ontology for General Medical Science (OGMS) [29] is a high-level OBO Foundry ontology for representing entities involved in health care encounters, such as diseases and their manifestations. OGMS (see Figure 3) extends the Basic Formal Ontology (BFO) [30] to represent a **disease**^[32]^ as a type of BFO **disposition**^[33]^, a **disorder**^[34]^as a type of BFO **material entity**^[35]^, and a **disease course**^[36]^ as a type of BFO **process**^[37]^.

**Figure 4:**
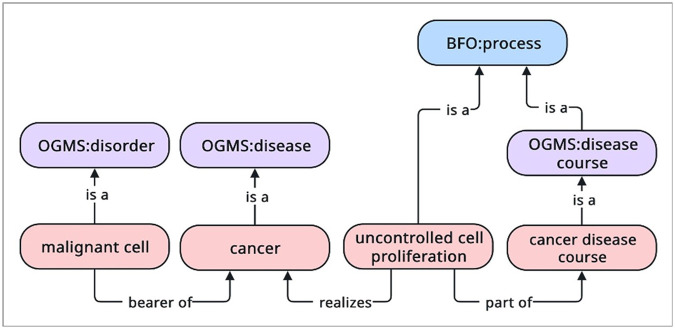
An example of OGMS’ account of disorder and disease. The malignant cell bears the disease of cancer, and the process of uncontrolled cell proliferation realizes cancer. The process of uncontrolled cell proliferation is part of the larger cancer disease course.

Colloquially speaking, a disposition, in this sense, is a property of a physical object that is exhibited when the object is engaged in certain kinds of processes. For example, the fragility (a property) of glass (a physical object) is a disposition that is exhibited when glass is shattering (a process). Within the context of OGMS, a **disease** is a propensity of an anatomical entity to engage in pathological behavior, a **disorder** is the physical entity that serves as a *bearer of* a **disease**, and the **process** during which one or more disease-related pathological behaviors unfold is a **disease course**. For example, cancer as a type of **disease** is a **disposition** of cells to proliferate uncontrollability, invade surrounding tissues, and metastasize to other sites of the body. A malignant cell that engages in these pathological behaviors is a type of *disorder*, and a process (such as uncontrolled cell proliferation) that *realizes*one or more of these pathological behaviors are part of cancer’s **disease course** (see Figure 4).

This distinction between **disease, disorder**, and **disease course** stands in contrast to SNOMED CT, ICD-11, MeSH, and NCIt, which often gloss over these distinctions. OGMS does not have terms for dental caries or dysbiosis, but OGMS’ representation of **disease** as a type of **disposition** has had significant influence on how DO, MONDO, the OHD represent diseases such as dental caries.

#### 3.5.2 Human Disease Ontology (DO)^[38]^

The Human Disease Ontology (DO) is an OBO Foundry ontology to classify human diseases for the purpose of facilitating better data integration and sharing of research findings. DO follows OGMS’ model of defining a **disease**^[39]^ (although the identifier is different), and is defined as a type of disposition:

A disease is a disposition (i) to undergo pathological processes that (ii) exists in an organism because of one or more disorders in that organism.

We note that the term ‘disposition’ is not explicitly defined in DO. However, since DO’s definition of disease is the same as OGMS’ definition of disease, we find it is reasonable to hold that DO’s use of the word ‘disposition’ refers to OGMS’ disposition

**Figure 5:**
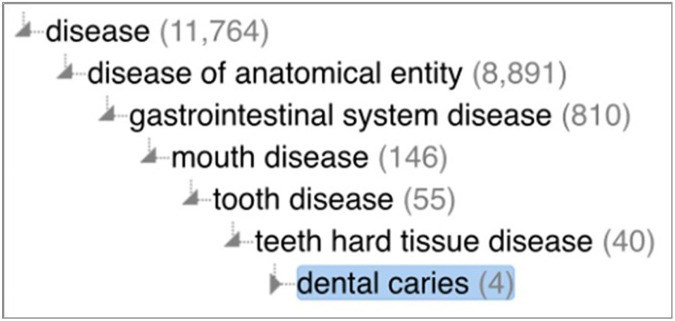
The **dental caries** hierarchy in the Human Disease Ontology.

In DO, **dental caries**^[40]^is defined as a type of **disease** (see Figure 5):

A teeth hard tissue disease that is characterized by damage to a tooth that can happen when decay-causing bacteria in your mouth make acids that attack the tooth’s surface, or enamel.

Further subtypes of **dental caries**, such **enamel caries**^[41]^ and **dentin caries**^[42]^ are also defined based on where the caries is located.

Unfortunately, the DO is not adequate for representing dental caries in two ways. First, DO does not contain concepts for the disorders, such as carious lesions, that are formed by the processes in which **dental caries** (the **disposition**) is exhibited. If the classification of **dental caries** as a **disposition** is strictly adhered to, it is not clear how useful the DO is to clinicians. Clinicians observe the physical damage, such as demineralized tooth surfaces, caused by excessive acid production, not the disposition to produce acids that cause demineralization.

Second, DO’s definition of **dental caries** specifies that it, loosely speaking, is a property of a tooth’s hard tissues. However, this is not correct. The excess acid production is the result of imbalances within the microbiome, not the tooth. Moreover, since DO does not contain concepts for representing dysbiosis or the oral microbiome, it is not possible for DO to accurately represent dental caries.

#### 3.5.3 Monarch Disease Ontology (MONDO)^[43]^

**Figure 6:**
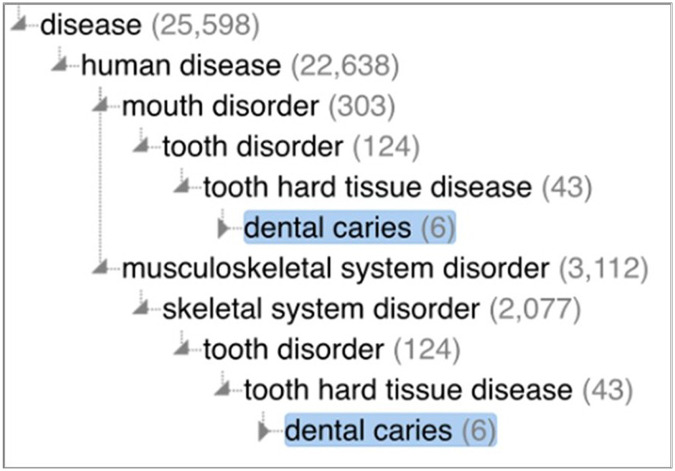
The **dental caries** hierarchy in the Monarch Disease Ontology.

The Monarch Disease Ontology (MONDO) is an OBO Foundry ontology that classifies diseases for the Monarch Initiative, a collaborative effort aimed at integrating and harmonizing data related to human diseases, phenotypes, and genotypes. In MONDO, **dental caries**^[44]^ is classified as both a **mouth disorder**^[45]^ and **skeletal system disorder**^[46]^ (see Figure 6).

Despite the use of the word ‘disorder’ in their names, **mouth disorder** and **skeletal system disorder** are defined as diseases.

mouth disorder def= A disease involving the mouth.

skeletal system disorder def= A disease involving the skeletal system.

MONDO’s classification of **dental caries** is lacking in several ways. First, MONDO’s definition of **dental caries** connotes it as being a process of tooth decay and not a disposition:

The decay of a tooth, in which it becomes softened, discolored, and/or porous.

Since MONDO definition of **disease**^[47]^ is almost identical to the DO definition, we find MONDO’s definition of **dental caries** inconsistent with its classification hierarchy. In other words, **dental caries** cannot be both a **disposition** and a **process**. Moreover, even if we grant a dispositional interpretation of MONDO’s definition, it still suffers from the aforementioned inadequacies of DO’s classification of dental caries.

Second, MONDO classified **dental caries** as a type of **human disease**^[48]^. Since many other types of animals, such as dogs, also experience caries, this classification is not correct. Finally, similar to DO, MONDO lacks concepts for representing disorders resulting from excess acid production and imbalances in the oral microbiome.

#### 3.5.4 Human Phenotype Ontology (HPO)^[49]^

The Human Phenotype Ontology (HPO) is an ontology designed to classify human disease phenotypes with the aim of providing a structured framework for organizing and integrating phenotype information between databases. Unlike DO and MONDO, HPO represents **Carious teeth**^[50]^(not dental caries) as a kind of bacterial infection:

Caries is a multifactorial bacterial infection affecting the structure of the tooth. This term has been used to describe the presence of more than expected dental caries.

**Figure 7:**
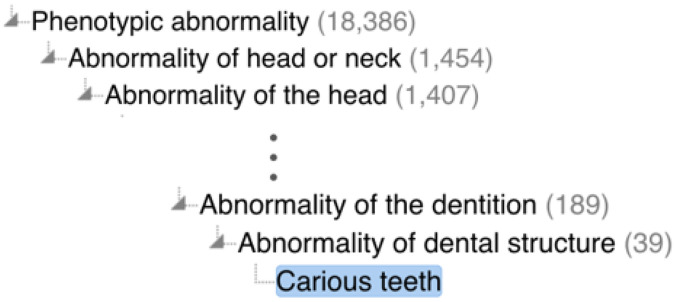
The **Carious teeth** hierarchy in the Human Phenotype Ontology.

While we agree with the lexical definition of caries being a bacterial infection, we find HPO inadequate as an ontological representation of dental caries for several reasons. First, the definition of **Carious teeth** is not congruent with the formal taxonomic structure of HPO in which **Carious teeth** is a type of **Abnormality of dental structure**^[51]^: An abnormality of the structure or composition of the teeth (see Figure 7).

HPO’s definition of **Carious teeth** states that it affects the structure of the teeth rather than being an abnormality of the structure or composition of the teeth (themselves). Moreover, the definition of **Carious teeth** is confusing about whether there is a distinction between caries and dental caries. In the definition, caries (the infection) has been used to describe the presence of dental caries. So, does **Carious teeth** represent the visible dental caries (i.e., abnormal dental structures) or does it represent the infection?

Finally, like DO and MONDO, HPO does not have concepts for representing the oral microbiome and oral microbial dysbiosis. If HPO was extended to include these concepts, one possibility is to define oral microbial dysbiosis as a type of **Abnormal homeostasis**^[52]^, and the acids resulting from the dysbiosis as a type of **Abnormality of acid-base homeostasis**^[53]^. However, it is not clear how to relate these concepts using HPO’s framework. The relations in HPO refer to concepts such as material entities and processes. For example, the *produces*^[54]^ relation is defined as:

a produces b if some process that occurs_in a has_output b, where a and b are material entities. Examples: hybridoma cell line produces monoclonal antibody reagent; chondroblast produces avascular GAG-rich matrix.

However, neither **Abnormal homeostasis** nor **Abnormality of acid-base homeostasis** are classified as being material entities or processes. In other words, the formal structure of HPO’s **Phenotypic abnormality**^[55]^ branch is not adequate to use relations such as *produces*.

#### 3.5.5 Oral Health and Disease Ontology (OHD)

The Oral Health and Disease Ontology (OHD) is an ontology for the dental domain and can be used to annotate dental electronic records with terms for dental conditions and interventions used to manage such conditions. The OHD adheres to the OGMS model of representing diseases as dispositions, and previous versions of OHD defined **dental caries**^[56]^ as:

A disease realized as a disease course in which a carious lesion develops in a tooth, resulting in demineralization, loss of tooth structure/appearance of a cavity or other structural damage to the tooth.

Unlike DO and MONDO, this definition does not ontologically commit caries to be a disease of tooth (or hard tissue) per se. However, it does explicitly relate **dental caries** to oral microbial dysbiosis. To rectify this, we made the following changes to the OHD.

First, to represent the disordered nature of microbiome dysbiosis, we imported the terms **infection**^[57]^, **infectious disorder**^[58]^, **infectious disease**^[59]^, and **infectious disease course**^[60]^from the Infectious Disease Ontology (IDO) [31].

**infection** =df A part of an extended organism that itself has as part a population of one or more infectious agents and that (1) exists as a result of processes initiated by members of the infectious agent population and is (2) clinically abnormal in virtue of the presence of this infectious agent population, or (3) has a disposition to bring clinical abnormality to immunocompetent organisms of the same Species as the host (the organism corresponding to the extended organism) through transmission of a member or offspring of a member of the infectious agent population.

**infectious disorder** =df An infection that is clinically abnormal.

**infectious disease** =df A disease whose physical basis is an infectious disorder.

**infectious disease course** =df A disease course that is the realization of an infectious disease.

**Figure 8:**
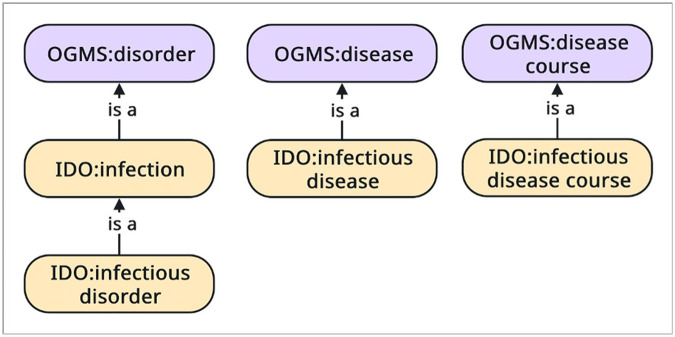
The hierarchy of the **infectious disorder, infectious disease**, and **infectious disease course** in the Infectious Disease Ontology.

In IDO, **infection** and **infectious disorder** are a type of OGMS **disorder**. This classification (see Figure 8) lays the foundation for extending the OHD to represent dysbiosis in the oral microbiome.

Second, using IDO’s terms and structure, we created the term **oral microbial dysbiosis**^[61]^in the OHD as a type of **infectious disorder** and redefined **dental caries** as a type of **infectious disease**.

**oral microbial dysbiosis** =df An infectious disorder that is the result of an imbalance or disruption of the microorganisms which normally exist within the mouth.

**dental caries** =df An infectious disease that is realized in a disease course during which cariogenic microbiota in oral biofilm produce acids that are capable of demineralizing dental hard tissues.

To relate **dental caries** to **oral microbial dysbiosis**, we then use the relation *disease has basis in dysfunction of*^[62]^ imported from the Relation Ontology (RO) [32].

*disease has basis in dysfunction of* =df A relation that holds between the disease and a material entity where the physical basis of the disease is a disorder of that material entity that affects its function.

**Figure 9:**
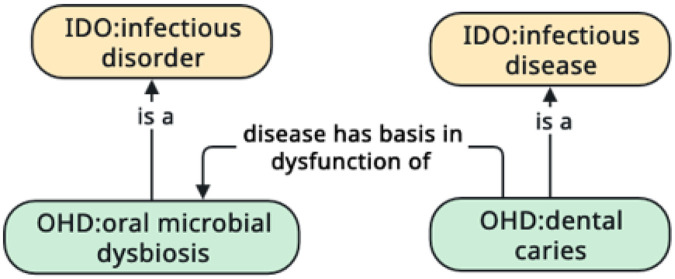
The representation of **oral microbial dysbiosis** in the Oral Health and Disease Ontology.

That is, we represent the physical basis **oral microbial dysbiosis** has for **dental caries** using the OWL [33] axiom **dental caries** *disease has basis in dysfunction of* some **oral microbial dysbiosis** (see Figure 9).

Third, to provide an account for a **carious tooth lesion**^[63]^, a type of **tooth disorder**^[64]^, that may result from dysbiotic activities within the oral microbiome, we created the terms **dental caries disease course**^[65]^(a type of **infectious disease course)** and **bacterial tooth demineralization process**^[66]^, with **dental caries disease course** as a type of IDO **infectious disease course** and **bacterial tooth demineralization** as type of OGMS **pathological bodily process**^[67]^.

**tooth disorder** =df A disorder that is part of a tooth.

**carious tooth lesion** =df A tooth disorder that affects a tooth that includes both the infectious organisms, the material they generate from the tooth, any immune effectors that are a response to the presence of the disorder and the physical changes to the tooth (i.e., demineralization or a cavity) resulting from the disorder.

**dental caries disease course** =df An infectious disease course that realizes dental caries.

**pathological bodily process** =df A bodily process that is clinically abnormal.

**bacterial tooth demineralization process** =df A pathological bodily process during which tooth mineral is solubilized by acid produced by certain bacteria that adhere to the tooth surface in bacterial communities known as dental plaque.

Formally, we relate **dental caries disease course, bacterial tooth demineralization process**, and **carious tooth lesion** using RO’s *part of*^[68]^and *output of*^[69]^ relations as follows:

**bacterial tooth demineralization process** *part of* some **dental caries disease course**

**carious tooth lesion** *output of* some**bacterial tooth demineralization process**

**Figure 10:**
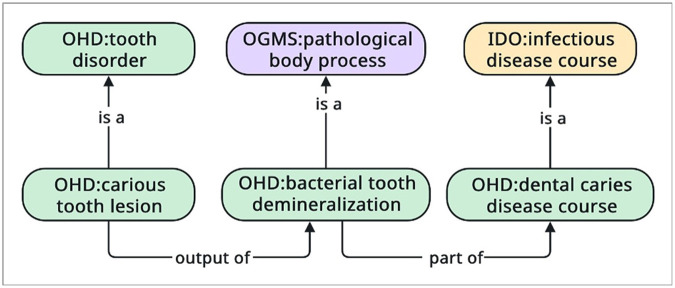
In the Oral Health and Disease Ontology, a **carious tooth lesion** is the output of (or the result of) **bacterial tooth demineralization**, which is part of the larger **dental caries disease course**.

In other words, the production of acids by bacteria that demineralize tooth structure occur as part of (or within) the larger set of cariogenic-disease processes, and the tooth cavities (i.e., lesions) are an outcome of the demineralization process (see Figure 10).

To represent the interaction between the dysbiotic activities within the oral microbiome and the **dental caries disease course**, we incorporate RO’s *causally influences*^[70]^relation.

*causally influences* =df The entity or characteristic A is causally upstream of the entity or characteristic B, A having an effect on B. An entity corresponds to any biological type of entity as long as a mass is measurable. A characteristic corresponds to a particular specificity of an entity (e.g., phenotype, shape, size).

Formally, this interaction is defined using the OWL axiom **oral microbial dysbiosis** *causally influences* some **dental caries disease course**.

Finally, we define the term **oral microbiome**^[71]^ as a subtype of the Ontology of Host-Microbiome Interactions (OHMI) [34] **microbiome**^[72]^ and Environment Ontology (ENVO) [35] **biome**^[73]^ terms.

oral microbiome =df A microbiome that is located in the mouth.

The relationship between **oral microbial dysbiosis** and the **oral microbiome** is represented using RO’s *part of* relation: **oral microbial dysbiosis** *part of* some **oral microbiome**.

**Figure 11:**
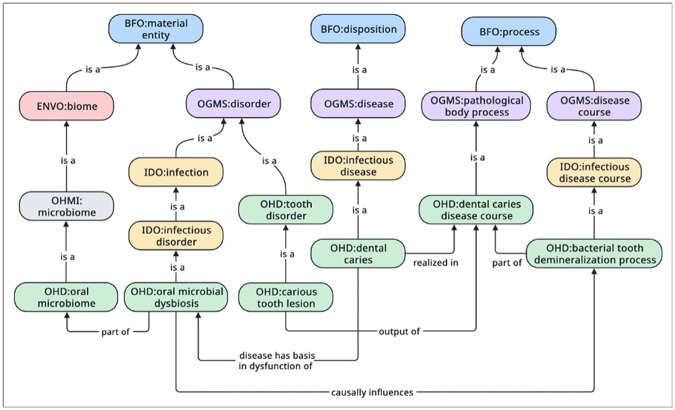
The complete representation of how **dental caries** is related to **oral microbial dysbiosis** in the Oral Health and Disease Ontology.

Figure 11 depicts the relation between **oral microbial dysbiosis, oral microbiome,** and **dental caries disease course** and provides a complete picture of OHD’s representation of **dental caries**.

## 4. Discussion

In recent years, there has been a paradigm shift in cariology. It now stresses the complexity of the development and progression of the disease with many conjoint elements that depend largely on host-microbe interaction [36]. Furthermore, treatment for dental caries has shifted from a surgical-restorative model to a preventive and disease management-based model [37].

Our review revealed broad shortcomings across existing ontologies and terminologies, underscoring their inability to fully capture the multifactorial nature of dental caries. Clinical classification systems like SNOMED CT and ICD-11, for example, define caries strictly as a lesion or disorder of tooth hard tissues, with no links to the oral microbiome or biofilm dysbiosis. This narrow, anatomy-centric focus means key etiological factors – such as microbial imbalances, dietary sugars, or omits saliva-mediated effects. Research vocabularies like MeSH and NCIt likewise exhibit conceptual inconsistencies, often conflating the disease process with its outcomes (e.g. treating *“dental caries”* as both the decay process and the cavity lesion) or using ambiguous definitions that blur whether caries is a pathological process or a physical structure. Meanwhile, disease ontologies in the OBO Foundry (including DO, MONDO, and HPO) adopt a formal disease model (inspired by OGMS) but still lack explicit representations for oral biofilm dysbiosis, acid-driven demineralization processes, or the resulting tooth lesions. In their current state, none of these resources can represent the full etiologic network of caries – they cannot relate a dysbiotic microbiome to caries onset, nor incorporate the myriad risk and protective factors that modulate disease progression. Additional host and environmental variables (for instance, high-sugar diets, medications, smoking, saliva flow, or even stress) are largely ignored [38], even though such factors can aggravate or attenuate dysbiosis and enamel degradation.

These gaps highlight an urgent need for ontologies to evolve beyond static lesion descriptors. Future ontology development should integrate host–microbe–environment interactions and acknowledge the spectrum from healthy homeostasis to dysbiosis [39]. Notably, a consensus in cariology now views dental caries as a “community-scale metabolic disorder” driven by complex microbial communities rather than a single pathogen [40]. This perspective implies that ontologies must be flexible and extensible – capable of linking dysbiotic states to downstream pathological processes, modeling preventative or aggravating factors, and accommodating new discoveries – so that our representations of oral health keep pace with emerging scientific understanding.

The OHD’s framework representing dental caries is a significant advance over current ontologies and terminologies. However, there are still areas that need to be addressed. To fully represent the etiology of oral microbial dysbiosis, it is necessary to represent the factors that impact the host-microbiome interaction. These include, among others, how host-derived factors, such as medication history, smoking status, and diet, interact with (or impact) the oral microbiome environment. Moreover, we have not included particular species of microbes often found in the oral microbiome. Adding terms for these microbes as well the particular functions they serve within the microbiome is potential future enhancement of the OHD.

## 5. Conclusion

In this paper, we demonstrated that current medical ontologies and terminologies have not kept pace with the recent shift in understanding the etiology of caries. Instead, they remain focused on the development or existence of lesions and lack the expressiveness to describe the role of biofilm ecology and microbiome imbalance during the caries disease course. These deficiencies motivated us to restructure the OHD to include terms that represent oral microbial dysbiosis and disease processes that arise from an imbalanced microbiome. This restructuring is advantageous for two reasons. First, it aligns the OHD with modern cariology. Keeping pace with scientific advances is critical for ensuring that an ontology’s terms remain relevant to the scientific community, and, as a result, the OHD is an advance over the aforementioned ontologies. Second, the OHD provides a semantic framework for including more dysbiosis-related terms, such as specific kinds of oral microbial dysbiosis and dysbiosis treatment, and, hence, can more easily incorporate new findings in the field of cariology.

## Footnotes

[1] Searches for articles and ontologies were conducted on 2025-07-04.

[2] The analysis is based on the 2025-06-01 release of SNOMED CT.

[3] SCTID: 116680003

[4] SCTID: 64572001

[5] SCTID: 40468400

[6] SCTID: 80967001

[7] SCTID: 312128007

[8] SCTID: 46557008

[9] SCTID: 427898007

[10] SCTID: 1162599006

[11] SCTID: 1071000119107

[12] SCTID: 1366725006

[13] SCTID: 716305005

[14] SCTID: 1149492007

[15] DA08.0

[16] DA08

[17] The analysis is based on the content in MESH as of 2025-02-17.

[18] ID D003731, http://id.nlm.nih.gov/mesh/D003731

[19] ID D017001, http://id.nlm.nih.gov/mesh/D017001

[20] ID D064806, http://id.nlm.nih.gov/mesh/D064806

[21] ID D010335, http://id.nlm.nih.gov/mesh/D010335

[22] ID D009057, http://id.nlm.nih.gov/mesh/D009057

[23] https://www.ncbi.nlm.nih.gov/mesh/68003731, accessed 2025-02-17

[24] https://www.nlm.nih.gov/mesh/intro_entry.html, accessed 2025-02-17

[25] https://www.ncbi.nlm.nih.gov/mesh/68064806, accessed 2025-02-17

[26] The analysis is based on the 2025-05-27 release of NCIt.

[27] Code C52593, https://evsexplore.semantics.cancer.gov/evsexplore/hierarchy/ncit/C52593

[28] Code C60925, https://evsexplore.semantics.cancer.gov/evsexplore/hierarchy/ncit/C60925

[29] Code C66861, https://evsexplore.semantics.cancer.gov/evsexplore/hierarchy/ncit/C66861

[30] C125208, https://evsexplore.semantics.cancer.gov/evsexplore/concept/ncit/C125208

[31] The analysis is based on the 2021-08-19 release of OGMS.

[32]
http://purl.obolibrary.org/obo/OGMS_0000031

[33]
http://purl.obolibrary.org/obo/BFO_0000016

[34]
http://purl.obolibrary.org/obo/OGMS_0000045

[35]
http://purl.obolibrary.org/obo/BFO_0000040

[36]
http://purl.obolibrary.org/obo/OGMS_0000063

[37]
http://purl.obolibrary.org/obo/BFO_0000015

[38] The analysis is based on the 2025-02-02 release of DO.

[39]
http://purl.obolibrary.org/obo/DOID_4

[40]
http://purl.obolibrary.org/obo/DOID_216

[41]
http://purl.obolibrary.org/obo/DOID_217

[42]
http://purl.obolibrary.org/obo/DOID_10461

[43] The analysis is based on the 2025-02-04 release of MONDO.

[44]
http://purl.obolibrary.org/obo/MONDO_0005276

[45]
http://purl.obolibrary.org/obo/MONDO_0006858

[46]
http://purl.obolibrary.org/obo/MONDO_0005172

[47]
http://purl.obolibrary.org/obo/MONDO_0000001

[48]
http://purl.obolibrary.org/obo/MONDO_0700096

[49] The analysis is based on the 2025-01-16 release of HPO.

[50]
http://purl.obolibrary.org/obo/HP_0000670

[51]
http://purl.obolibrary.org/obo/HP_0011061

[52]
http://purl.obolibrary.org/obo/HP_0012337

[53]
http://purl.obolibrary.org/obo/HP_0004360

[54]
http://purl.obolibrary.org/obo/RO_0003000

[55]
http://purl.obolibrary.org/obo/HP_0000118

[56]
http://purl.obolibrary.org/obo/OHD_0000031

[57]
http://purl.obolibrary.org/obo/IDO_0000586

[58]
http://purl.obolibrary.org/obo/IDO_0000504

[59]
http://purl.obolibrary.org/obo/IDO_0000436

[60]
http://purl.obolibrary.org/obo/IDO_0000495

[61]
http://purl.obolibrary.org/obo/OHD_0000420

[62]
http://purl.obolibrary.org/obo/RO_0004020

[63]
http://purl.obolibrary.org/obo/OHD_0000021

[64]
http://purl.obolibrary.org/obo/OHD_0000260

[65]
http://purl.obolibrary.org/obo/OHD_0000416

[66]
http://purl.obolibrary.org/obo/OHD_0000417

[67]
http://purl.obolibrary.org/obo/OGMS_0000061

[68]
http://purl.obolibrary.org/obo/BFO_0000050

[69]
http://purl.obolibrary.org/obo/RO_0002353

[70]
http://purl.obolibrary.org/obo/RO_0002566

[71]
http://purl.obolibrary.org/obo/OHD_0000418

[72]
http://purl.obolibrary.org/obo/OHMI_0000002

[73]
http://purl.obolibrary.org/obo/ENVO_00000428

## Abbreviations

Ontologies and terminologies

BFO: Basic Formal Ontology
DO: Human Disease Ontology
HPO: Human Phenotype Ontology
ICD-11: The International Classification of Diseases, 11th ed.
IDO: Infectious Disease Ontology
ENVO: Environment Ontology
MeSH: Medical Subject Headings
MONDO: Monarch Disease Ontology
NCIt: NCI Thesaurus
OGMS: Ontology for General Medical Science
OHD: Oral Health and Disease Ontology
OHMI: Ontology of Host-Microbiome Interactions
OWL: Web Ontology Language
RO: Relation Ontology
SNOMED CT: Systematized Nomenclature of Medicine - Clinical Terms
